# The indirect effects of smartphone addiction on accidental injuries: a cross-sectional study among medical college students

**DOI:** 10.3389/fpubh.2025.1695798

**Published:** 2026-01-12

**Authors:** Baifeng Chen, Yuan Xiao, Rui Wang, Yu Zhu, Yali Liang, Lei Ding

**Affiliations:** 1 School of Public Health, Wannan Medical College, Wuhu, China; 2 Wuhu Center for Disease Control and Prevention, Wuhu Health Supervision Bureau, Wuhu, China

**Keywords:** accidental injuries, delayed bedtime, distracted walking, physical activity, smartphone addiction

## Abstract

**Background:**

Campus pedestrian injury risks are often overlooked. While existing literature focuses on direct links between specific smartphone-related behaviors (e.g., distracted walking) and accidental injuries, the indirect mechanisms through which smartphone addiction influences such injuries remain unclear. This study investigates and quantifies these modifiable behavior-mediated indirect pathways to inform more targeted injury prevention strategies.

**Methods:**

A cross-sectional survey of 1,235 university students in 2023 assessed demographic characteristics, accidental injuries, smartphone addiction, and smartphone-related behaviors. Multivariable logistic regression identified injury risk factors, and path analysis examined indirect relationships between smartphone addiction and accidental injuries.

**Results:**

The incidence of accidental injuries was higher in the smartphone addiction group (20.9%) than in the non-addiction group (13.2%). Logistic regression showed that bedtime smartphone use, physical inactivity, and smartphone-engaged walking increased injury risk (*p* < 0.05). Path analysis indicated that smartphone addiction indirectly increased injury risk through these behaviors, accounting for 57.7% of the total effect (*β* = 0.105, 95% CI: 0.08–0.13).

**Conclusion:**

Smartphone addiction significantly increases the risk of accidental injuries among university students, primarily mediated by behaviors such as bedtime smartphone use, smartphone-engaged walking, and physical inactivity. Targeted interventions addressing smartphone use could reduce injuries in this population.

## 1 Introduction

University life is a critical developmental stage marked by academic engagement and increased independence. However, it also carries a heightened risk of accidental injuries. Recent studies have shown that college students experience high rates of collisions, slips, and falls on campuses (1). In China, rapid expansion of higher education has led to crowded campuses, where high pedestrian density and limited space contribute to injury risk (2). Unlike off-campus environments dominated by traffic accidents, campus injuries are typically related to walking collisions, falls, or slips in pedestrian-heavy areas (3).

Accidental injuries among university students are multifactorial, resulting from the interaction of behavioral, psychological, and environmental factors. Behavioral factors like distracted walking (4), physical inactivity (5), and insufficient sleep (6) are known to increase susceptibility to injuries. Psychological factors, such as academic pressure and anxiety, may amplify these risks (7). The widespread use of smartphones in students’ lives has added a new behavioral dimension to this issue. Smartphones have transformed communication, learning, and entertainment (8), but excessive use can lead to smartphone addiction (SA)—a behavioral dependence that disrupts functioning (9). Related phenomena, such as nomophobia and fear of missing out, are specific manifestations of problematic smartphone use, reflecting the broader spectrum of smartphone dependence. These subtypes of smartphone-related disorders have been conceptualized as distinct expressions of maladaptive smartphone use, with recent studies highlighting their psychological and behavioral correlates (10, 11).

Smartphone addiction is particularly prevalent among university students and has been linked to multiple negative outcomes, including anxiety, depression, sleep deprivation, and physical inactivity (8, 12–14). A cross-sectional study among Turkish adolescents found that 46.9% exhibited symptoms of SA, with over 90% reporting phone use before sleep (15). While research has primarily focused on the psychological consequences of SA, its contribution to accidental injuries remains underexplored (16). Previous studies have shown that phone use while walking impairs situational awareness and increases the likelihood of collisions (17–19); however, it remains unclear whether the risk persists after accounting for other smartphone-related behaviors. Moreover, emerging evidence suggests that SA may influence multiple domains of behavior simultaneously—such as delayed bedtime, reduced physical activity, and distracted ambulation (13, 14, 20, 21)—which may collectively contribute to injury risk. However, the mechanisms through which SA influences accidental injuries remain unclear.

To address these gaps, the present study investigated the relationship between SA and accidental injuries among Chinese university students, focusing on the indirect effects mediated by smartphone-related behaviors, including bedtime smartphone use, smartphone-engaged walking, and physical inactivity. By identifying these behavioral pathways, the study aims to provide a theoretical and empirical foundation for targeted injury prevention strategies in university settings.

## 2 Materials and methods

### 2.1 Study design and population

This cross-sectional study was conducted in October 2023 among undergraduate students at Wannan Medical College, excluding fifth-year medical students due to clinical internships. A total of 1,330 participants were randomly selected using stratified cluster sampling based on academic year and class. Of the completed self-administered questionnaires, 1,235 valid responses were obtained (92% response rate). Based on pre-defined criteria, 107 questionnaires were excluded due to: (1) missing values for key variables or >20% of demographic variables; or (2) logical inconsistencies (e.g., reporting no smartphone use while completing the SAS-SV). Data cleaning was performed using Epi Info 3.1, and a random 5% sample of excluded questionnaires was independently verified by two researchers to ensure consistency. Non-response analysis showed no significant differences in gender or grade level (*p* > 0.05), indicating minimal selection bias.

### 2.2 Demographic and physical activity

The demographic section collected information on participants’ gender, age, academic standing, residential background (urban or rural), monthly living expenses, and physical activity frequency. The study used a revised version of Liang’s Physical Activity Rating Scale (22), which demonstrated good reliability (Cronbach’s *α* = 0.821) based on Meng et al.’s (23) research. Physical activity (PA) levels were assessed using a 5-point Likert scale (1–5 for frequency, duration, and intensity). The physical activity score was calculated as: frequency × (duration – 1) × intensity. Total scores ranged from 0 to 100, with participants stratified into three groups: low-level PA (≤19), moderate-level PA (20–42), or high-level PA (≥43).

### 2.3 Smartphone addiction scale

Mobile phone addiction was assessed using the Smartphone Addiction Scale-Short Version (SAS-SV), as used in our prior research (24). The SAS-SV is a validated scale consisting of 10 items rated on a 6-point scale (1 “strongly disagree” to 6 “strongly agree”). Total scores range from 10 to 60, with higher scores indicating greater smartphone addiction in the past year. The SAS-SV demonstrated content and concurrent validity, as well as internal consistency (Cronbach’s *α* = 0.91). Cut-off values of ≥31 for males and ≥33 for females, as suggested by Kwon et al. (25), were applied to determine addiction status.

### 2.4 Unhealthy smartphone-related behaviors

The “Unhealthy smartphone-related behaviors” scale was developed through a multi-step process involving literature review, expert consultation, and pilot testing. Ten candidate items were generated based on previous studies on problematic smartphone use (26, 27). After review by three public-health experts and two behavioral scientists, six items with the highest content relevance and clarity were retained. A pilot test with 80 undergraduate students assessed reliability and item comprehensibility. The pilot demonstrated good internal consistency (Cronbach’s *α* = 0.732) and test-retest reliability over a two-week interval (*r* = 0.78), indicating satisfactory consistency and stability.

In the formal survey, respondents rated the six behaviors that occurred in the past 12 months on a 5-point Likert scale (1 = never to 5 = every day): (a) academic procrastination attributable to smartphone use, (b) missing transit stops during smartphone engagement, (c) delayed sleep onset due to nocturnal smartphone use, (d) diminished in-person social interactions due to excessive device use, (e) smartphone-engaged walking, and (f) smartphone use during cycling. Responses were dichotomized using a cutoff of ≥3, coded as “yes.” This procedure ensured that the items were both culturally appropriate and psychometrically reliable for the target population.

### 2.5 Accidental injuries

Based on Kim et al.’s (27) study, we developed a scale to assess smartphone-related injuries. Participants were asked to report occurrences of two predefined accident types: (a) falls or slips, and (b) bumps or collisions. A sample item was: “Have you experienced any fall or slip-related injuries in the past 12 months?” Participants who reported at least one such incident were classified as having experienced smartphone-related injuries, with responses coded dichotomously (yes/no).

### 2.6 Statistical analysis

We first conducted descriptive analyses to characterize the study population. To identify factors associated with accidental injuries among college students, we performed univariate analyses using chi-square tests, followed by multivariate binary logistic regression. Consistent with established methodological guidelines, we included all variables showing marginal associations (*p* < 0.20) in univariate analyses as potential candidates for multivariate modeling. Stepwise backward elimination was applied to retain only variables significant at *p* < 0.05, reporting adjusted odds ratios (aORs) with corresponding 95% confidence intervals. To further examine the complex relationships among variables, we constructed a path analysis model in AMOS software based on theoretical frameworks and logistic regression results, aiming to quantify both direct and indirect effects of significant factors on accident risk. The path model specifically tested the hypothesis that smartphone addiction (SA) influences injury risk indirectly through behavioral mediators rather than through direct effects. All data were managed using Epi Info 3.1 and analyzed in SPSS 26.0 (IBM Corp., Armonk, NY, United States), with statistical significance set at *α* = 0.05 (two-tailed).

## 3 Results

### 3.1 Basic information of the research subject

The basic characteristics of the participants are shown in Table 1. The study included 1,235 undergraduate students aged 17–26 years (mean age ± SD = 19.80 ± 1.47 years). Of these, 529 (42.8%) were male, and 706 (57.2%) were female. The sample included 334 freshmen (27.0%), 272 sophomores (22.0%), 337 juniors (27.3%), and 292 seniors (23.6%). In terms of geographical background, 739 students (59.8%) were from rural areas, and 496 (40.2%) were from urban areas. Physical activity levels were categorized as follows: low-level (567 participants, 45.9%), moderate-level (550 participants, 44.5%), and high-level (118 participants, 9.6%). Additionally, 63.9% (*n* = 789) of the participants met the criteria for SA. The incidence of accidental injuries was 18.1% among the study participants.

**Table 1:** Basic information of the participants.

Characteristics	Number of cases (*n*)	Proportion (%)
Age group		
≤18 years	243	19.7
19–20 years	557	45.1
≥21 years	435	35.2
Sex		
Male	529	42.8
Female	706	57.2
Student grade level		
Grade 1	334	27.0
Grade 2	272	22.0
Grade 3	337	27.3
Grade 4	292	23.6
Residential source		
Rural areas	739	59.8
Urban areas	496	40.2
Monthly living expenses (CNY)		
<1,200	439	35.5
1,200–1,500	476	38.5
>1,500	320	25.9
Physical activity		
Low-level	567	45.9
Moderate-level	550	44.5
High-level	118	9.6
Smartphone addiction		
Positive	789	63.9
Negative	446	36.1
Accidental injuries		
Yes	224	18.1
No	1,011	81.9

### 3.2 Univariate analysis of the association between smartphone addiction and unhealthy smartphone-related behaviors among college students

As shown in Table 2, univariate analysis revealed that SA was significantly associated with several adverse behavioral outcomes, including impaired academic performance (OR = 3.38, 95% CI: 2.40–4.77), increased late-night device usage (OR = 1.71, 95% CI: 1.26–2.33), reduced social engagement (OR = 1.96, 95% CI: 1.50–2.57), and decreased physical activity (low-level vs. high-level: OR = 1.65, 95% CI: 1.10–2.47). Additionally, it increased situational risks such as missing transit stops (OR = 1.62, 95% CI: 1.04–2.52), distracted walking (OR = 1.44, 95% CI: 1.07–1.95), and cycling (OR = 1.56, 95% CI: 1.19–2.06), ultimately raising vulnerability to accidental injuries (OR = 1.73, 95% CI: 1.26–2.40).

**Table 2:** Univariate analysis of the association between college students’ smartphone addiction and unhealthy phone usage habits.

Variables	SA positive [*n* (%)]	SA negative [*n* (%)]	*x* ^2^	*p*	OR (95% CI)
Decline in academic performance					
Yes	221 (82.8)	46 (17.2)	52.66	<0.001	3.38 (2.40–4.77)
No	568 (58.7)	400 (41.3)			Ref.
Bedtime smartphone use					
Yes	685 (65.9)	354 (34.1)	11.83	0.001	1.71 (1.26–2.33)
No	104 (53.1)	92 (46.9)			Ref.
Reduced social engagement					
Yes	281 (74.1)	98 (25.9)	24.93	<0.001	1.96 (1.50–2.57)
No	508 (59.3)	348 (40.7)			Ref.
Physical activity					
Low-level	392 (69.1)	175 (30.9)	5.87	0.018	1.65 (1.10–2.47)
Moderate-level	352 (64.0)	198 (36.0)	1.69	0.208	1.31 (0.87–196)
High-level	68 (57.6)	50 (42.4)			Ref.
Smartphone-related missed bus stops					
Yes	80 (73.4)	29 (26.6)	4.68	0.036	1.62 (1.04–2.52)
No	709 (63.0)	417 (37.0)			Ref.
Smartphone-engaged walking					
Yes	670 (65.4)	355 (34.6)	5.72	0.017	1.44 (1.07–1.95)
No	119 (56.7)	91 (43.3)			Ref.
Smartphone-engaged cycling					
Yes	237 (71.2)	96 (28.8)	10.48	0.001	1.56 (1.19–2.06)
No	552 (61.2)	350 (38.8)			Ref.
Accidental injuries					
Yes	165 (73.7)	59 (26.3)	11.33	0.001	1.73 (1.26–2.40)
No	624 (61.7)	387 (38.3)			Ref.

### 3.3 Univariate analysis of risk factors for accidental injuries among university students

As shown in Table 3, univariate analysis revealed statistically significant associations between accidental injuries and several risk factors. Mobile phone dependency was associated with a higher likelihood of accidental injuries (OR = 1.73, 95% CI: 1.26–2.40). Prolonged phone use before sleep also increased injury risk (OR = 1.80, 95% CI: 1.14–2.86). Notably, phone use while walking posed the highest risk (OR = 2.68, 95% CI: 1.62–4.45), followed by phone use during cycling (OR = 1.98, 95% CI: 1.46–2.68). Additionally, physical inactivity was associated with a modest but significant increase in injury risk (low-level vs. high-level: OR = 1.89, 95% CI: 1.09–3.28).

**Table 3:** Univariate analysis of accidental injuries and several risk factors among college students.

Variables	No accidental injuries [*n* (%)]	Accidental injuries [*n* (%)]	*x* ^2^	*p*	OR (95% CI)
Student grade level					
Grade 1 and Grade 2	490 (80.9)	116 (19.1)	0.81	0.369	0.88 (0.66–1.17)
Grade 3 and Grade 4	521 (82.8)	108 (17.2)			Ref.
Sex					
Male	442 (83.6)	87 (16.4)	1.78	0.182	1.22 (0.91–1.65)
Female	569 (80.6)	137 (19.4)			Ref.
Residential source					
Rural areas	605 (81.9)	134 (18.1)	0.00	0.996	1.00 (0.75–1.35)
Urban areas	406 (81.9)	90 (18.1)			Ref.
Monthly living expenses (CNY)					
<1,500	750 (82.0)	165 (18.0)	0.03	0.872	1.03 (0.74–1.43)
>1,500	261 (81.6)	59 (18.4)			Ref.
SA					
Positive	624 (79.1)	165 (20.9)	11.33	0.001	1.73 (1.26–2.40)
Negative	387 (86.8)	59 (13.2)			Ref.
Bedtime smartphone use					
Yes	838 (80.7)	201 (19.3)	6.43	0.011	1.80 (1.14–2.86)
No	173 (88.3)	23 (11.7)			Ref.
Smartphone-engaged walking					
Yes	819 (79.9)	206 (20.1)	15.60	<0.001	2.68 (1.62–4.45)
No	192 (91.4)	18 (8.6)			Ref.
Smartphone-engaged cycling					
Yes	246 (73.9)	87 (26.1)	19.60	<0.001	1.98 (1.46–2.68)
No	765 (84.8)	137 (15.2)			Ref.
Physical activity					
Low-level	430 (75.8)	137 (24.2)	5.33	0.021	1.89 (1.09–3.28)
Moderate-level	451 (82.0)	99 (18.0)	0.87	0.422	1.30 (0.75–2.28)
High-level	101 (85.6)	17 (14.4)			Ref.

### 3.4 Multivariate logistic analysis of factors associated with accidental injuries among college students

As shown in Table 4, multivariable binary logistic regression analysis identified several independent risk factors significantly associated with accidental injuries among college students. The final model revealed that pre-sleep excessive smartphone use (aOR = 1.68, 95% CI: 1.06–2.69) and physical inactivity (aOR = 1.40, 95% CI: 1.03–1.90) were significant predictors. Most notably, smartphone use while walking had the strongest association (aOR = 7.17, 95% CI: 2.61–19.69), followed by smartphone use during cycling (aOR = 1.59, 95% CI: 1.11–2.29).

**Table 4:** Logistic regression analysis of risk factors for accidental injuries in the study population.

Variables	*B*	S.E.	Wals	Sig	aOR (95% CI)
Bedtime smartphone use (yes vs. no)	1.21	0.4	9.19	0.028	1.68 (1.06–2.69)
Physical activity (high-level = 1, moderate-level = 2, low-level = 3)	0.33	0.19	6.61	0.033	1.40 (1.03–1.90)
Smartphone-engaged walking (yes vs. no)	1.97	0.52	14.58	<0.001	7.17 (2.61–19.69)
Smartphone-engaged cycling (yes vs. no)	0.47	0.19	6.29	0.012	1.59 (1.11–2.29)

aOR, Adjusted odds ratio; CI, Confidence interval.

Multivariate binary logistic regression model adjusted for age, sex, residential, and monthly cost.

### 3.5 Path analysis of the relationship between smartphone addiction and accidental injuries among college students

**Figure 1:**
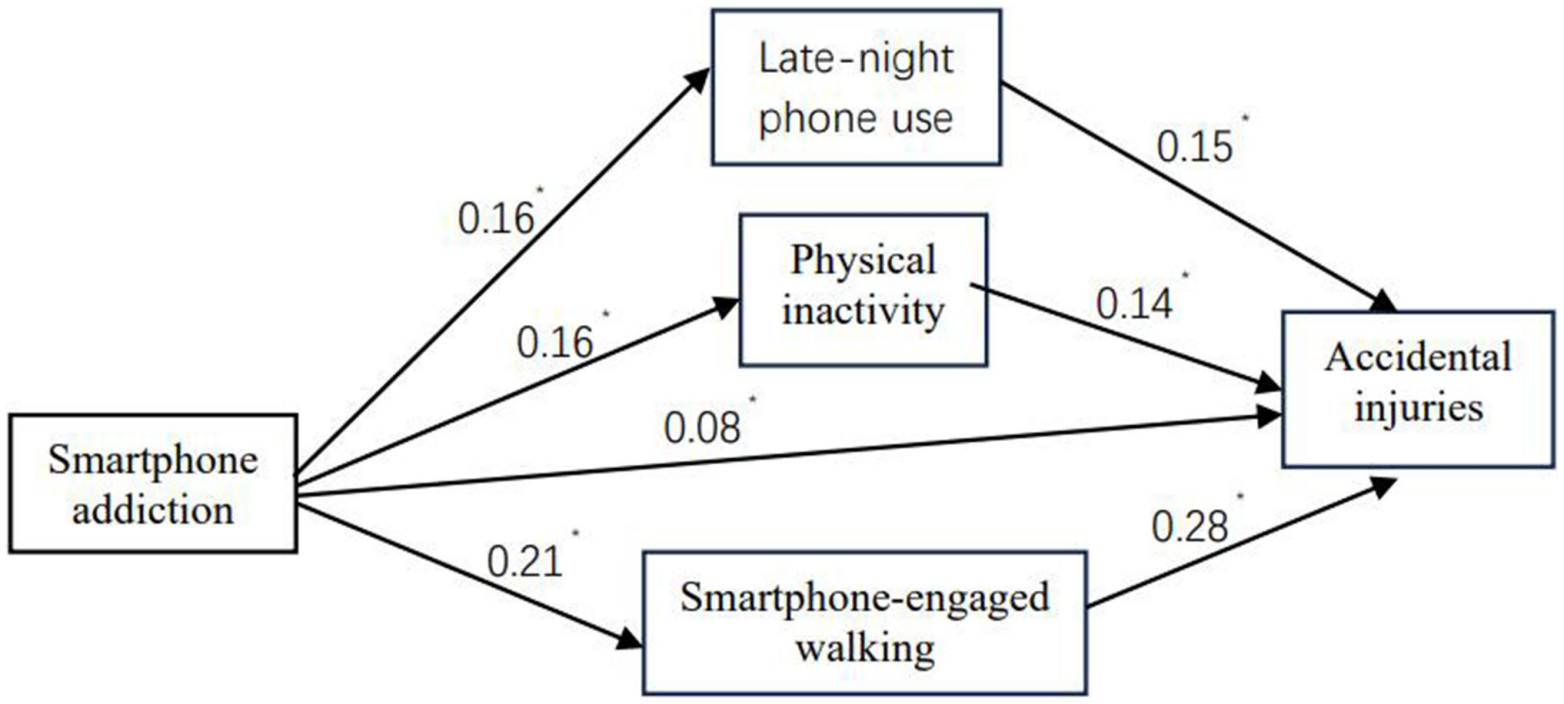
Model designed for examination of the relations between smartphone addiction and accidental injuries. ^*^p < 0.01, two-tailed.

To further examine the mechanisms underlying the association between SA and accidental injuries, a path analysis was conducted. The results are presented in Figure 1 and Table 5. The final model demonstrated good fit to the data (NFI = 0.96, IFI = 0.97, CFI = 0.96, RMSEA = 0.056, RMR = 0.031, *χ*^2^/d*f* = 4.93). Significant direct effects on accidental injuries were observed for bedtime smartphone use (*β* = 0.15), physical inactivity (*β* = 0.14), smartphone-engaged walking (*β* = 0.28), and SA (*β* = 0.08). Importantly, SA also exhibited a significant indirect effect (*β* = 0.105, 95% CI: 0.08–0.13) through three behavioral mediators: bedtime smartphone use, smartphone-engaged walking, and physical inactivity. These mediating variables accounted for 57.7% of the total effect of SA on accidental injuries.

**Table 5:** Results of the mediation analysis examining the effect of smartphone addiction on accidental injuries.

Variables	*β*	*β* (95% CI)	Proportion (%)	S.E.	*p*
Indirect effect: SA → bedtime smartphone use → accidental injuries	0.024	0.014–0.034	13.1	0.005	<0.001
Indirect effect: SA → physical inactivity → accidental injuries	0.022	0.012–0.032	12.0	0.005	<0.001
Indirect effect: SA → smartphone-engaged walking → accidental injuries	0.059	0.043–0.075	32.3	0.008	0.001
Total indirect effect via three paths	0.105	0.083–0.129	57.7	0.011	<0.001
Direct effect: SA → accidental injuries	0.077	0.028–0.126	42.3	0.025	0.005
Total effect	0.182	0.132–0.233	100.0	0.025	<0.001

## 4 Discussion

### 4.1 Summary and interpretation of main findings

This study found that SA significantly increased the risk of accidental injuries among university students. The overall incidence of accidental injuries was 18.1%, with a notably higher rate among students with SA (20.9%) compared to those without (13.2%), consistent with previous reports from Chinese and Korean universities (27, 28). Path analysis showed that 57.7% of the total effect of SA on accidental injuries was mediated through three behavioral factors: bedtime smartphone use, smartphone-engaged walking, and physical inactivity. Although prior studies (29, 30) have shown sex- and age-related differences in walking-related risks and smartphone distractions, such demographic variations were not observed in our university sample. This may reflect the relatively homogeneous age and sex distribution of university students.

These findings suggest that the association between SA and accidental injuries is mediated through behavioral factors. Rather than being a simple consequence of excessive device usage, the risk arises primarily from maladaptive habits that accompany addiction—particularly distracted walking, delayed bedtime, and sedentary behaviors. Previous studies have emphasized distraction as a dominant mechanism (16, 18). Our study differs from previous research in measurement tools, study populations, and analytic approach, extending this understanding by quantifying the relative contributions of multiple modifiable behaviors. This behavioral mediation model underscores that SA affects physical safety through intertwined lifestyle changes rather than a single risk behavior (21, 31).

### 4.2 Smartphone-engaged walking and situational distraction

Smartphone-engaged walking emerged as the strongest behavioral mediator (*β* = 0.28), consistent with prior research showing that smartphone distraction during walking reduces situational awareness and reaction time, increasing the likelihood of collisions and falls (4, 17–19). Studies indicate that 15–35% of pedestrians use smartphones while walking or crossing roads, with young adults being particularly vulnerable (4, 32, 33). In congested campus environments where walking is the primary mode of transportation (3), even brief lapses in attention can lead to near-miss or injury incidents.

Mechanistically, phone use competes for cognitive resources, narrowing peripheral vision and impairing environmental scanning (19). Our findings confirm that smartphone-engaged walking is a key behavioral pathway linking SA to injury risk. From a public health perspective, interventions such as safe-walking education, smartphone-free zones, and visual reminders may reduce preventable campus injuries among students with high smartphone dependency.

### 4.3 Bedtime smartphone use, sleep disturbance, and injury vulnerability

Bedtime smartphone use was another significant mediator in the SA-injury relationship. Consistent with previous research (34–36), nighttime device use delays sleep onset and reduces sleep quality, leading to daytime fatigue and impaired concentration. Sleep deprivation decreases attention, balance, and motor control (37–39), increasing the likelihood of unintentional injuries during daily activities.

In our model, bedtime smartphone use accounted for approximately 13.1% of the total mediated effect. This suggests that poor sleep patterns represent a behavioral vulnerability linking SA to physical injury. Promoting sleep hygiene and discouraging smartphone use before bedtime could therefore mitigate injury risk. University-based health programs incorporating digital behavior management and sleep education may provide a feasible strategy for reducing injury incidence among students.

### 4.4 Physical inactivity and diminished motor resilience

Physical inactivity also mediated the relationship between SA and accidental injuries, accounting for approximately 12% of the total effect. Consistent with prior studies, SA often displaces time spent on physical activity, leading to sedentary lifestyles (13, 20, 21). Reduced physical activity weakens muscle strength, coordination, and balance, all of which increase susceptibility to slips and falls (5, 40).

This behavioral mechanism illustrates how SA indirectly undermines physical resilience. Inactive individuals have slower neuromuscular responses and diminished stability, which can lead to minor but recurrent injuries. Therefore, promoting regular physical activity as part of digital health interventions could improve both physical and mental well-being, counteracting the adverse effects of excessive smartphone use.

### 4.5 Public health implications and limitations

These findings show that 57.7% of the total effect of SA on accidental injuries is mediated by modifiable behaviors—smartphone-engaged walking, bedtime smartphone use, and physical inactivity. This suggests that more than half of the injury risk associated with SA could be prevented through behavioral change. From a public health perspective, integrating smartphone use management with sleep hygiene promotion and physical activity programs could form the basis of campus injury-prevention strategies. Clinicians and educators could also benefit from screening for SA and delivering brief interventions to encourage safer and healthier smartphone behaviors.

However, several limitations should be acknowledged. First, the study participants were recruited from a single medical university, which limits the generalizability of the findings to other regions and academic disciplines. Second, our analysis focused on on-campus incidents and did not include off-campus or traffic-related injuries. Third, other unmeasured variables—such as psychological stress or environmental hazards—may also contribute to injury risk. Fourth, data collected only in October fail to capture seasonal variations in SA (e.g., more indoor use in extreme weather) and injury incidence (e.g., winter ice-related slips), which may alter the associations between SA, mediating behaviors, and injuries and thus compromise external validity. Finally, the cross-sectional design precludes causal inference. Future longitudinal and multi-site studies are warranted to validate these findings and to explore additional behavioral and psychosocial mediators.

## 5 Conclusion

In conclusion, smartphone addiction significantly increases university students’ risk of accidental injuries, primarily mediated by three modifiable behaviors: smartphone-engaged walking, bedtime smartphone use, and physical inactivity. Collectively, these mediators explain over half of the total effect of smartphone addiction (SA) on injury risk. These findings underscore that intermediate behaviors are the key drivers of SA-associated injury risk, addressing these behavioral pathways via digital wellness interventions, sleep hygiene education, and physical activity promotion could substantially reduce unintentional injuries while enhancing students’ overall health and safety.

## Data Availability

The raw data supporting the conclusions of this article will be made available by the authors, without undue reservation.
